# Diagnostic Intervals and Its Association with Breast, Prostate, Lung and Colorectal Cancer Survival in England: Historical Cohort Study Using the Clinical Practice Research Datalink

**DOI:** 10.1371/journal.pone.0126608

**Published:** 2015-05-01

**Authors:** Maria Theresa Redaniel, Richard M. Martin, Matthew J. Ridd, Julia Wade, Mona Jeffreys

**Affiliations:** 1 NIHR CLAHRC West, University of Bristol, Bristol, United Kingdom; 2 School of Social and Community Medicine, University of Bristol, Bristol, United Kingdom; University of Campinas, BRAZIL

## Abstract

Rapid diagnostic pathways for cancer have been implemented, but evidence whether shorter diagnostic intervals (time from primary care presentation to diagnosis) improves survival is lacking. Using the Clinical Practice Research Datalink, we identified patients diagnosed with female breast (8,639), colorectal (5,912), lung (5,737) and prostate (1,763) cancers between 1998 and 2009, and aged >15 years. Presenting symptoms were classified as alert or non-alert, according to National Institute for Health and Care Excellence guidance. We used relative survival and excess risk modeling to determine associations between diagnostic intervals and five-year survival. The survival of patients with colorectal, lung and prostate cancer was greater in those with alert, compared with non-alert, symptoms, but findings were opposite for breast cancer. Longer diagnostic intervals were associated with lower mortality for colorectal and lung cancer patients with non-alert symptoms, (colorectal cancer: Excess Hazards Ratio, EHR >6 months vs <1 month: 0.85; 95% CI: 0.72-1.00; Lung cancer: EHR 3-6 months vs <1 month: 0.87; 95% CI: 0.80-0.95; EHR >6 months vs <1 month: 0.81; 95% CI: 0.74-0.89). Prostate cancer mortality was lower in patients with longer diagnostic intervals, regardless of type of presenting symptom. The association between diagnostic intervals and cancer survival is complex, and should take into account cancer site, tumour biology and clinical practice. Nevertheless, unnecessary delay causes patient anxiety and general practitioners should continue to refer patients with alert symptoms via the cancer pathways, and actively follow-up patients with non-alert symptoms in the community.

## Introduction

Between 1995 to 2007, the five-year relative survival from breast, colorectal, lung and prostate cancers was 7 to 10% lower in the UK compared with other developed countries [1–3]. This was attributed to delayed diagnosis or presentation at a very advanced stage [2,4]. Rapid diagnostic pathways and targets were implemented within the UK National Health Service (NHS), with the aim of improving cancer outcomes and increasing cancer survival in the UK [5,6]. The National Institute for Health and Care Excellence (NICE) also set out referral guidelines to expedite the referral of patients with symptoms that are directly suggestive of cancer [7,8].In the cancer patient pathway, primary care and referral delays account for a bigger proportion of the total delay in days compared to secondary care delay [9]. Shortening diagnostic intervals (time between presentation to health care professionals and diagnosis) was made a priority as part of the early diagnosis initiative by the UK Department of Health [10]. Shorter diagnostic intervals may result to earlier tumour stage at diagnosis, which could then lead to improved outcomes, including survival [11,12]. However, evidence shows that the effect of diagnostic intervals on survival differs by cancer site [11–23], with some studies suggesting that longer diagnostic intervals are associated with higher mortality for cancers of the urinary tract, colon and breast [11,22,24], while others variously report an absence of any association for breast, colorectal, lung and gastro-oesophageal cancers [13,15,18,22,24], higher mortality with shorter diagnostic intervals for lung cancers [19,20], or higher mortality with both shorter and longer diagnostic intervals for colorectal cancer (i.e. a U-shaped relationship) [21].This variation in associations between diagnostic interval and survival may reflect differences in clinical detection pathways, patient and physician behaviour, functioning of the health care system, and the biological behaviour of the tumour [13]. The role of tumour aggressiveness has been suggested to explain some of the counter-intuitive findings, where patients with longer diagnostic intervals show better survival compared to patients with shorter diagnostic intervals (waiting time paradox) [12]. More aggressive tumours are likely to cause symptoms that would draw attention to the underlying cancer and may prompt earlier diagnosis, but would also spread rapidly and result in poorer prognosis [16,21,24]. Slow growing tumours produce non-definitive symptoms that could lead to longer diagnostic interval [16,21], but would also allow time for treatment [24]. Given this potential for ‘confounding by indication’, symptom presentation should be considered in analyses of associations between diagnostic interval and survival. Using an historical cohort of patients with breast, colorectal, lung and prostate cancer, we assessed associations of diagnostic interval (time from presentation in primary care to diagnosis) with five-year survival, and stratified these by NICE-qualifying alert and non-alert symptom presentations [7,8].

## Materials and Methods

### Data sources

Data for this analysis came from the Clinical Practice Research Datalink (CPRD, formerly General Practice Research Database or GPRD), a large computerized database of anonymised primary care medical records [26]. It currently includes prospectively gathered administrative, clinical and prescribing records for about 5 million active patients from over 600 primary practices throughout the UK, equating to 7% of the population [26]. Individuals registered on the database are representative of the age, sex and geographical distribution of the UK population. Data are subject to thorough validation, audit and quality checks [26,27] and there is a high level of diagnostic validity for cancers [25,28]. The CPRD records were linked to National Cancer Data Repository (NCDR) and to the 2007 English Indices of Multiple Deprivation (IMD) datasets. These linkages were only performed for records from general practices in England who agreed to such linkages (about 52% of CPRD practices). The NCDR captures data from the Merged Cancer Registry (containing clinical and tumour characteristics), the inpatient Hospital Episode Statistics (HES, source of ethnicity data) and the Office of the National Statistics (ONS, source of mortality data) [29]. The IMD dataset contains the 2007 IMD score [30], which was used in the study as an indicator of the level of deprivation (see below). The linked datasets were provided to the researchers in an anonymised form.The databases cover different time periods (CPRD: 1996–2009; Merged Cancer Registry: 1996–2009; HES: 1997–2011; ONS: 1998–2010) and analyses were limited to patients diagnosed in the years covered by all four datasets (1998–2009).

### Study population

From all patients who were registered in the CPRD, patients diagnosed with female breast, colorectal, lung and prostate cancers between January 1, 1998 and December 31, 2009, who were 15 years and older at the time of diagnosis were included in the study. The cohort was defined as patients registered in the Merged Cancer Registry as having a primary tumour classified as C50 (breast), C18-C20 (colorectal), C34 (lung) and C61 (prostate) in the International Classification of Diseases (ICD 10). Patients diagnosed with secondary cancers, in situ cancers or diagnosed via death certificates (DCO) or autopsy only were excluded.From a total of 62,178 English cancer patients who were eligible in the CPRD dataset, 45,766 (73%) were linked to the Merged Cancer Registry, HES and ONS datasets. Of these, 22,051 (48%) presented to a GP with a cancer symptom one year prior to diagnosis. The distribution of patients who were excluded from the analysis were mostly similar to patients who were included (data not shown). Breast cancer patients in the CPRD but not linked to the other datasets had a slightly higher proportion of patients aged 15–44. Breast and colorectal cancer patients in the cancer registry records but not found in the CPRD had fewer patients with poorly and undifferentiated cancers.

### Study variables

Diagnostic interval was defined as the time between the date of presentation in primary care and the date of cancer diagnosis. This interval was categorised a priori as <1 week, 1–2 weeks, 3–4 weeks and >1 month for breast cancer, and <1 month, 1–2 months, 3–6 months and >6 months for colorectal, lung and prostate cancer, based on time intervals that the clinical researchers (MR and RM) considered meaningful to clinical practice. We used different categories for the time intervals for breast cancer as 75% of patients were seen within 31 days of symptom presentation compared to 130 days or more for the other cancer sites.A primary care presentation was defined as the earliest date of consultation with a general practitioner (GP), occurring up to one year prior to the first cancer diagnosis, where a patient was recorded by the GP as having either a NICE-qualifying alert or non-alert symptom. A NICE-qualifying alert symptom was defined as a symptom suggestive of cancer, and requires urgent referral based on the NICE guidelines [7]. A non-alert symptom was defined as a symptom suggestive of low risk, but predictive of cancer, and was based on symptoms reported in the published literature [31]. Symptoms were site-specific and the classifications were agreed by clinical researchers and epidemiologists (MR, RM and MTR). We have excluded investigations such as PSA testing and chest x-ray, as these will be preceded by symptoms that would have led to the investigation. The age criterion was applied for symptoms of colorectal cancer with a qualifying age. For symptoms with a qualifying duration (for example, persistent or present for a number of weeks) and description (for example, hard), we assumed that the symptoms fulfilled the criterion. The date of diagnosis was defined as the date of the first event or event of higher priority appearing in the patients’ medical records (if recorded within three months of the first event) among the following, in order of priority: histological or cytological confirmation, admission to the hospital or first consultation at the outpatient clinic because of the malignancy [32]. All definitions were in line with the Aarhus checklist for early cancer-diagnosis research [33].Survival was defined as the number of days between the date of diagnosis and the date of outcome (death or censoring). Follow-up was censored at 5 years, as is commonly practiced in population-based cancer survival studies, or at the end of the study period (December 31, 2009), whichever came earlier. Other variables included in the analysis were age, sex (for colorectal and lung cancers), ethnicity, region of residence, level of deprivation, period of cancer plan implementation, stage and tumour subsite (for colorectal cancer), tumour differentiation, morphology, tobacco smoking status (for lung cancer), comorbidity, treatment and number of consultations prior to symptom presentation. Age at cancer diagnosis was categorized as 15–44, 45–54, 55–64, 65–74 and 75 years and above. Due to the small numbers in the 15–44 age group, the first two age groups were combined for prostate cancer. Ethnicity was self-reported, as recorded in the HES database [34,35], and was categorized as White, Black, Asian, mixed, and other ethnic group. Geographical region was defined as the patient’s region of residence at the time of diagnosis.Level of deprivation was based on the IMD. It is calculated for small geographical areas known as Lower Super Output Areas (LSOAs), each of which is comprised of a minimum population of 1000 and an average of 1500 [36], based on patients’ area of residence at the time of diagnosis. It is comprised of a number of social and economic indicators (housing, employment, income, access to services, education and skills, crime, and living environment) [30]. Quintiles based on English 2007 IMD scores were computed, with the first quintile designated as the least deprived. To account for changes in clinical practice brought about by the Cancer Plan (2000), we controlled for the implementation period of the waiting time targets. This was defined in the Cancer Plan as prior to implementation (1996–2000), initialization (breast cancer: 2001–2002; other sites: 2001–2005) and implementation (breast cancer: 2002–2009; other sites: 2006–2009). Staging for colorectal cancer was based on the Dukes’ Classification, as TNM staging was not available in the databases. There was no or very limited staging information available for the other cancer sites and this variable was not included in the analysis for those sites. Tumour differentiation was defined as well-, moderately-, poorly- and un-differentiated. Tumour morphology was based on the International Classification of Diseases for Oncology (ICD-O-3) and was specific for the cancer site: breast: invasive ductal carcinoma (8500), invasive lobular carcinoma (8520) and other types [37]; colorectal: adenocarcinoma (8140), mucinous adenocarcinoma (8480) and other types [38]; lung: squamous cell carcinoma (8070, 8052, 8084, 8073, 8083), small cell carcinoma (8041, 8045), adenocarcinoma (8140, 8255, 8550, 8260, 8250, 8252, 8253, 8254, 8230, 8333, 8480, 8470, 8490, 8310), large cell carcinoma (8012, 8013, 8123, 8082, 8310, 8014) and other types [39]; prostate: adenocarcinoma (8140) and other types [40]. Tumour subsite (for colorectal cancer) was colon, rectosigmoid or rectum.Tobacco smoking status (specific for lung cancer patients) was defined as the last status recorded prior to cancer diagnosis, classified as non-smoker, current smoker and ex-smoker. Comorbidity was measured using the CPRD-based Charlson Comorbidity Index [41], derived for each patient for comorbid conditions occurring in the one year period prior to the date of cancer diagnosis, and categorised as 0, 1 or 2 or more co-morbid conditions. Treatment was included as a proxy variable for stage, and refers to surgery, radiotherapy, chemotherapy and hormone therapy. Each treatment regimen was treated as an individual variable, coded as no treatment, received treatment or unknown. The number of consultations prior to presentation is the total number of consultations within the one year period prior to the date of the first presentation with a symptom of cancer (either NICE-qualifying alert or non-alert). This number was used a proxy for propensity to consult with a clinician, to take into account increased likelihood of being diagnosed with each additional consultation, and was categorised as 0, 1–2, 3–5, 6–10 or more than 10.

### Data Analysis

Relative survival (RS) is a measure of survival that accounts for mortality due to causes other than cancer. It is the ratio of the observed survival of cancer patients to the probability of survival that would have been expected if patients had the same survival probability as the general population [42]. Estimates of relative survival were stratified by the nature of the symptoms (NICE-qualifying alert and non-alert) and were computed using the complete approach (where all patients diagnosed between 1998 and 2009 were included, regardless of whether they had full five-year or partial follow-up) [43]. These estimates were expressed as percentages and were computed using the STRS command in STATA. Survival probabilities were estimated at intervals of 6 months in the first year, then yearly up to 5 years. We used age-, sex-, region- and deprivation specific single-year life tables [44] to account for differences in the underlying mortality, and used the Ederer II method [42] to determine expected survival. We also estimated five-year relative survival, conditional on survival after 1 year to account for the effects of factors that strongly influence survival in the first year after diagnosis. This is to take into account the effect of stage, in the absence of a staging variable that could be used for breast, lung and prostate cancers. This is the cumulative four-year survival (at the fifth anniversary of diagnosis) for patients who were alive at the end of the first year. To determine the association between diagnostic interval and mortality, Excess Hazards Ratios (EHR) at five years were computed using a generalised linear model with a Poisson error structure [45]. The EHR is the ratio of mortality rates in the presence of one factor (e.g. White ethnicity) and the mortality rates in the absence of the same factor, once the reference population mortality is taken into account [45]. EHRs can be interpreted as equivalent to the mortality risk ratio. Univariable and multivariable models were built, specific for each cancer site, and stratified by the nature of the symptoms. Multivariable models controlled for the effects of age, sex (for colorectal and lung cancers), ethnicity, region of residence, level of deprivation, period of cancer plan implementation, stage and tumour subsite (for colorectal cancer), tumour differentiation, morphology, tobacco smoking status (for lung cancer), comorbidity, treatment and number of consultations prior to symptom presentation. We also tested for evidence of an interaction between diagnostic interval and age (with age as a binary variable, dichotomised at 60 years). We used the likelihood ratio test to determine goodness of fit.We employed multiple imputation using chained equations (ICE) to account for missing data on tumour differentiation, morphology, Dukes’ stage (colorectal cancer only), deprivation quintile, surgery, radiotherapy, chemotherapy and hormone therapy (Tables 1 and 2) [46,47]. Imputation models were derived for each missing variable and included: the exposure of interest (diagnostic interval); the incomplete variables; all other covariables; and outcome (survival time and outcome (dead or censored)). Since data for tumour differentiation was missing for more than half of the lung and prostate cancer patients, this variable was not imputed for these cancer sites, and the category of unknown was included in the analyses. A total of 20 complete datasets were constructed to reduce sampling variability from the imputation process [48] and the results were combined using Rubin’s Rules [46,47]. The distributions of the imputed variables were similar to the distributions of the measured variables. All regression analyses were based on the imputed dataset.All statistical analyses were carried out using STATA v12 software [49].

**Table 1:** Socio-demographic characteristics of patients by cancer site.

Variable	Breast		Colorectal		Lung		Prostate	
	N = 8,639		N = 5,912		N = 5,737		N = 1,763	
	Freq.	%	Freq.	%	Freq.	%	Freq.	%
**Age group (at diagnosis)**								
* 15–44*	1,537	17.8	151	2.6	83	1.4	2	0.1
* 45–54*	1,978	22.9	502	8.5	381	6.6	53	3.0
* 55–64*	1,556	18.0	1,222	20.7	1,285	22.4	338	19.2
* 65–74*	1,461	16.9	1,818	30.8	1,976	34.4	668	37.9
* 75 and above*	2,107	24.4	2,219	37.5	2,012	35.1	702	39.8
**Sex**								
* Male*	0	0.0	3,219	54.4	3,382	59.0	2,807	100.0
* Female*	8,639	100.0	2,693	45.6	2,355	41.0	0	0.0
**Ethnicity**								
* White*	6,636	76.8	4,736	80.1	4,323	75.4	1,347	76.4
* Black*	74	0.9	36	0.6	12	0.2	25	1.4
* Asian*	91	1.1	34	0.6	20	0.3	11	0.6
* Mixed*	14	0.2	6	0.1	1	0.0	2	0.1
* Other*	92	1.1	26	0.4	37	0.6	11	0.6
* Unknown*	1,732	20.0	1,074	18.2	1,344	23.4	367	20.8
**Region**								
* London*	929	10.8	565	9.6	558	9.7	184	10.4
* North East*	157	1.8	128	2.2	188	3.3	26	1.5
* North West*	1,424	16.5	1,007	17.0	1,247	21.7	278	15.8
* Yorkshire and the Humber*	466	5.4	284	4.8	331	5.8	90	5.1
* East Midlands*	339	3.9	284	4.8	247	4.3	62	3.5
* West Midlands*	1,174	13.6	797	13.5	728	12.7	212	12.0
* East of England*	1,127	13.0	773	13.1	696	12.1	243	13.8
* South East*	2,032	23.5	1,314	22.2	1,075	18.7	430	24.4
* South West*	991	11.5	760	12.9	667	11.6	238	13.5
**Level of Deprivation**								
* 1—least deprived*	2,242	26.0	1,391	23.5	904	15.8	500	28.4
* 2*	2,252	26.1	1,550	26.2	1,172	20.4	456	25.9
* 3*	1,666	19.3	1,208	20.4	1,134	19.8	350	19.9
* 4*	1,462	16.9	1,055	17.8	1,271	22.2	287	16.3
* 5—most deprived*	1,001	11.6	699	11.8	1,237	21.6	168	9.5
* Unknown*	16	0.2	9	0.2	19	0.3	2	0.1
**Period of Cancer Plan Implementation**								
* Prior to implementation*	1,348	15.6	847	14.3	927	16.2	217	12.3
* Initialization*	1,346	15.6	2,511	42.5	2,411	42.0	735	41.7
*Implementation*	5,945	68.8	2,554	43.2	2,399	41.8	811	46.0

**Table 2:** Tumour and clinical characteristics of patients by cancer site.

Variable	Breast		Colorectal		Lung		Prostate	
	Freq.	%	Freq.	%	Freq.	%	Freq.	%
**Symptoms at first presentation**								
* Alert*	8,150	94.3	2,178	36.8	533	9.3	357	20.2
* Non-alert*	489	5.7	3,734	63.2	5,204	90.7	1,406	79.8
**Tumour differentiation**								
* Well differentiated*	1,090	12.6	284	4.8	109	1.9	73	4.1
* moderately differentiated*	3,418	39.6	3,643	61.6	520	9.1	304	17.2
* poorly- or undifferentiated*	3,018	34.9	837	14.2	1,269	22.1	241	13.7
* unknown*	1,113	12.9	1,148	19.4	3,839	66.9	1,145	64.9
**Comorbidity**								
* 0*	7,818	90.5	5,053	85.5	4,467	77.9	1,473	83.6
* 1*	600	6.9	616	10.4	974	17.0	206	11.7
* 2 or more*	221	2.6	243	4.1	296	5.2	84	4.8
**Surgery**								
* no*	1,086	12.6	1,096	18.5	3,610	62.9	923	52.4
* yes*	7,288	84.4	4,552	77.0	1,343	23.4	635	36.0
* unknown*	265	3.1	264	4.5	784	13.7	205	11.6
**Radiotherapy**								
* no*	4,843	56.1	4,084	69.1	3,128	54.5	1,241	70.4
* yes*	2,849	33.0	895	15.1	2,028	35.3	275	15.6
* unknown*	947	11.0	933	15.8	581	10.1	247	14.0
**Chemotherapy**								
* no*	4,708	54.5	3,280	55.5	3,466	60.4	1,435	81.4
* yes*	2,790	32.3	1,891	32.0	1,658	28.9	44	2.5
* unknown*	1,141	13.2	741	12.5	613	10.7	284	16.1
**Hormone therapy**								
* no*	4,535	52.5	4,793	81.1	4,769	83.1	923	52.4
* yes*	3,094	35.8	15	0.3	62	1.1	646	36.6
* unknown*	1,010	11.7	1,104	18.7	906	15.8	194	11.0
**Number of consultations**								
* 0*	424	4.9	361	6.1	223	3.9	95	5.4
* 1–2*	2,083	24.1	1,321	22.3	1,098	19.1	326	18.5
* 3–5*	2,487	28.8	1,565	26.5	1,498	26.1	479	27.2
* 6–10*	2,161	25.0	1,598	27.0	1,586	27.6	510	28.9
* >10*	1,484	17.2	1,067	18.0	1,332	23.2	353	20.0
* median (IQR)*	5 (2–8)		5 (2–9)		6 (3–10)		5 (3–9)	

### Ethics approval

This project was approved by the NHS South Central—Berkshire B Research Ethics Board (11/SC/0387) and by the Faculty of Medicine and Dentistry Committee for Ethics (FCE), University of Bristol (101153). The use of data from the Clinical Practice Research Datalink was approved by the CPRD-Independent Scientific Advisory Committee (11_148**)**.

## Results

The final sample was comprised of 8,639 female breast, 5,912 colorectal, 5,737 lung and 1,763 prostate cancer patients. The distributions of the cases by the different socio-demographic, tumour and clinical characteristics are presented in Tables 1–2 and S1 Table. The majority (94.3%) of breast cancer patients presented with alert symptoms in the year prior to diagnosis, in contrast to 36.8% of colorectal, 9.3% of lung and 20.2% of prostate cancer patients (Table 2; S2–S5 Tables). Women with breast cancer had the shortest diagnostic intervals (median: 14 days; Interquartile range, IQR: 9–31; Table 3), while patients with lung cancer had the longest (median: 88; IQR: 34–210; Table 3). In all four cancer sites, patients who first presented with non-alert symptoms had longer diagnostic intervals than patients who first presented with alert symptoms (Table 3). Amongst breast cancer patients, 92.5% presented with a breast lump, an alert symptom, with a median diagnostic interval of 14 days (IQR: 9–28; S2 Table). The two most common presenting symptoms for colorectal cancer were abdominal pain (28.0%; median: 84; IQR: 33–175; S3 Table), a non-alert symptom, and rectal bleeding (20.7%; median: 47.5; IQR: 21–104), a NICE-qualifying symptom. For lung cancer, the most common presentations were for non-alert symptoms: cough (40.9%; median: 114; IQR: 44–241) and dyspnoea (18.5%; median: 71; IQR: 26–196; S4 Table). Nocturia, a non-alert symptom, was the most common presenting symptom for prostate cancer, reported by 37.2% of men (median: 71.5: IQR: 36–144; S5 Table).

**Table 3:** Characteristics of patients by diagnostic interval^¹^, symptom category and cancer site.

Cancer site / Diagnostic interval	All Patients		Alert symptoms		Non-alert symptoms	
	Freq.	%	Freq.	%	Freq.	%
**Breast**						
* < 1 week*	1,550	17.9	1,516	18.6	34	7.0
* 1–2 weeks*	3,013	34.9	2,957	36.3	56	11.5
* 3–4 weeks*	1,862	21.6	1,783	21.9	79	16.2
* > 1 month*	2,214	25.6	1,894	23.2	320	65.4
* Median^2^ (IQR* ³ *)*	14 (9–31)		14 (9–29)		55 (21–175)	
**Colorectal**						
* <1 month*	1,661	28.1	796	36.5	865	23.2
* 1–2 months*	1,156	19.6	510	23.4	646	17.3
* 3–6 months*	1,904	32.2	650	29.8	1,254	33.6
* >6 months*	1,191	20.1	222	10.2	969	26.0
* Median^2^ (IQR* ³ *)*	67 (27–147)		45 (20–95)		84 (34–186)	
**Lung**						
* <1 month*	1,304	22.7	236	44.3	1,068	20.5
* 1–2 months*	1,020	17.8	121	22.7	899	17.3
* 3–6 months*	1,707	29.8	122	22.9	1,585	30.5
* >6 months*	1,706	29.7	54	10.1	1,652	31.7
* Median^2^ (IQR* ³ *)*	88 (34–210)		35 (17–78)		99 (38–222)	
**Prostate**						
* <1 month*	374	21.2	113	31.7	261	18.6
* 1–2 months*	427	24.2	101	28.3	326	23.2
* 3–6 months*	649	36.8	101	28.3	548	39.0
* >6 months*	313	17.8	42	11.8	271	19.3
* Median^2^ (IQR* ³ *)*	71 (35–145)		48 (24–111)		76 (37–151)	

¹time between first symptom presentation in primary care and diagnosis

²number of days

³IQR: Interquartile range

### Relative survival

Five-year relative survival (RS) was highest for breast cancer, at 80.5% (95% Confidence Interval, 95% CI: 79.2–81.7%) and lowest for lung cancer with 7.9% (95% CI: 7.1–8.8%; Table 4). The five-year relative survival for colorectal and prostate cancers was 47.8% (95% CI: 46.0–49.5%) and 71.5% (95% CI: 67.8–75%), respectively. Stratified by the NICE-qualifying alert and non-alert symptoms, five-year relative survival for breast cancer was slightly higher in women who first presented with non-alert symptoms compared to those who presented with alert symptoms (RS non-alert: 85.8%; 95%CI: 81.0–89.8% vs. RS alert: 80.1; 95% CI: 78.9–81.4%). In contrast, the survival of patients with colorectal, lung and prostate cancer was greater in those who presented with NICE-qualifying alert, compared with non-alert, symptoms. Colorectal cancer patients who presented with NICE-qualifying symptoms had a 14.7 percentage points (95% CI: 13.9–15.4) higher five-year relative survival than patients who presented with non-alert symptoms (RS alert: 57.0%; 95% CI: 54.1–59.8% vs RS non-alert: 42.3%; 95% CI: 40.2–44.4%). The five-year survival of lung cancer patients who presented with alert symptoms was 4.6 percentage points (95% CI 2.0–7.2) higher compared to patients who presented with non-alert symptoms (RS alert: 12.0%; 95% CI: 9.4–15.1% vs RS non-alert: 7.4%; 95% CI: 6.6–8.3%). Prostate cancer men who presented with alert symptoms had a 5.8 percentage points (95% CI: 1.5–9.2) higher five-year relative survival compared to men who presented with non-alert symptoms (RS alert: 76.2%; 95% CI: 67.8–83.6% vs RS non-alert: 70.4%; 95% CI: 66.3–74.4%). The five-year relative survival estimates conditional on surviving the first year were all higher than the five-year relative survival estimates and followed the patterns of the relative survival estimates by symptom category (Table 5).

**Table 4:** Relative survival of cancer patients by site, diagnostic interval^¹^ and symptom category^²^.

Cancer site / Diagnostic Interval¹	All Patients				Alert Symptoms				Non-alert Symptoms			
	5-year Relative survival	95% Confidence Interval			5-year Relative survival	95% Confidence Interval			5-year Relative survival	95% Confidence Interval		
**Breast **												
* * **Overall**	**80.5**	**79.2**	**-**	**81.7**	**80.1**	**78.9**	**-**	**81.4**	**85.8**	**81.0**	**-**	**89.8**
* < 1 week*	78.4	75.1	**-**	81.5	78.2	74.8	**-**	81.3	88.2	64.3	**-**	100.0
* 1–2 weeks*	75.8	73.3	**-**	78.1	75.8	73.3	**-**	78.1	74.9	55.9	**-**	88.4
* 3–4 weeks*	84.0	81.5	**-**	86.4	84.3	81.8	**-**	86.7	76.2	60.2	**-**	87.7
* > 1 month*	84.2	82.0	**-**	86.2	83.4	81.0	**-**	85.6	89.4	84.0	**-**	93.6
**Colorectal**												
* * **Overall**	**47.8**	**46.0**	**-**	**49.5**	**57.0**	**54.1**	**-**	**59.8**	**42.3**	**40.2**	**-**	**44.4**
* <1 month*	44.0	40.7	**-**	47.2	51.6	46.7	**-**	56.3	36.9	32.7	**-**	41.2
* 1–2 months*	49.7	45.7	**-**	53.6	61.0	54.8	**-**	67.0	41.0	35.9	**-**	46.0
* 3–6 months*	51.7	48.7	**-**	54.6	63.1	57.9	**-**	68.0	45.6	42.0	**-**	49.2
* >6 months*	44.6	40.7	**-**	48.5	48.2	39.4	**-**	56.9	43.8	39.4	**-**	48.1
**Lung**												
* * **Overall**	**7.9**	**7.1**	**-**	**8.8**	**12.0**	**9.4**	**-**	**15.1**	**7.4**	**6.6**	**-**	**8.3**
* <1 month*	7.2	5.7	**-**	8.8	9.4	6.2	**-**	13.5	6.5	5.0	**-**	8.4
* 1–2 months*	6.9	5.2	**-**	8.8	13.2	8.0	**-**	20.1	5.7	4.1	**-**	7.7
* 3–6 months*	9.1	7.5	**-**	10.8	17.7	10.9	**-**	26.0	8.3	6.8	**-**	10.1
* >6 months*	7.9	6.5	**-**	9.6	9.8	3.4	**-**	20.7	7.9	6.4	**-**	9.6
**Prostate**												
* * **Overall**	**71.5**	**67.8**	**-**	**75.0**	**76.2**	**67.8**	**-**	**83.6**	**70.4**	**66.3**	**-**	**74.4**
* <1 month*	54.2	45.7	**-**	62.6	67.0	49.6	**-**	81.9	49.3	39.6	**-**	59.0
* 1–2 months*	70.6	62.7	**-**	78.0	77.2	61.0	**-**	90.0	68.7	59.5	**-**	77.2
* 3–6 months*	73.4	67.4	**-**	79.0	80.0	64.6	**-**	92.1	72.2	65.7	**-**	78.4
* >6 months*	85.9	77.9	**-**	92.8	82.4	58.4	**-**	98.6	86.4	77.8	**-**	94.0

¹time between first symptom presentation in primary care and diagnosis

²symptoms were categorized as alert or non-alert based on the NICE guidelines

**Table 5:** Conditional relative survival of cancer patients by site, diagnostic interval^¹^ and symptom category^²^.

Cancer site / Diagnostic intervals¹	All Patients				Alert symptoms				Non-alert symptoms			
	5-year Conditional Relative survival	95% Confidence Interval			5-year Conditional Relative survival	95% Confidence Interval			5-year Conditional Relative survival	95% Confidence Interval		
**Breast **												
* * **Overall**	**84.5**	**83.3**	**-**	**85.6**	**84.2**	**83.0**	**-**	**85.4**	**88.5**	**84.0**	**-**	**92.1**
* < 1 week*	83.0	79.9	**-**	85.8	82.8	79.7	**-**	85.7	93.6	70.0	**-**	102.6
* 1–2 weeks*	80.8	78.6	**-**	83.0	80.9	78.6	**-**	83.1	78.5	60.2	**-**	90.7
* 3–4 weeks*	87.4	85.0	**-**	89.5	87.7	85.3	**-**	89.8	79.8	64.6	**-**	90.2
* > 1 month*	87.0	85.0	**-**	88.9	86.3	84.0	**-**	88.3	91.7	86.6	**-**	95.3
**Colorectal**												
* * **Overall**	**67.1**	**64.9**	**-**	**69.2**	**71.2**	**67.9**	**-**	**74.4**	**64.0**	**61.2**	**-**	**66.9**
* <1 month*	63.5	59.2	**-**	67.6	66.7	60.8	**-**	72.1	59.6	53.3	**-**	65.7
* 1–2 months*	69.8	64.7	**-**	74.6	75.3	68.0	**-**	81.7	64.5	57.3	**-**	71.3
* 3–6 months*	69.6	66.0	**-**	73.0	75.7	70.0	**-**	80.9	65.6	60.9	**-**	70.1
* >6 months*	64.7	59.5	**-**	69.7	63.2	52.2	**-**	73.1	65.2	59.2	**-**	70.8
**Lung**												
* * **Overall**	**27.2**	**24.6**	**-**	**29.8**	**31.2**	**24.7**	**-**	**38.1**	**26.4**	**23.6**	**-**	**29.3**
* <1 month*	27.3	22.2	**-**	32.8	27.4	18.2	**-**	37.5	27.2	21.0	**-**	33.8
* 1–2 months*	25.2	19.3	**-**	31.5	34.0	20.8	**-**	48.3	22.7	16.3	**-**	29.9
* 3–6 months*	27.8	23.3	**-**	32.6	38.4	23.9	**-**	53.5	26.5	21.8	**-**	31.5
* >6 months*	27.4	22.5	**-**	32.5	24.9	8.1	**-**	47.3	27.6	22.6	**-**	33.0
**Prostate**												
* * **Overall**	**78.2**	**74.3**	**-**	**81.8**	**81.7**	**72.8**	**-**	**89.1**	**77.3**	**72.9**	**-**	**81.4**
* <1 month*	64.4	54.4	**-**	73.7	73.4	53.9	**-**	88.6	60.6	48.7	**-**	71.7
* 1–2 months*	75.7	67.2	**-**	83.1	81.9	64.4	**-**	94.4	73.8	64.0	**-**	82.5
* 3–6 months*	79.4	73.1	**-**	85.0	86.0	69.4	**-**	97.6	78.1	71.2	**-**	84.4
* >6 months*	91.8	83.6	**-**	98.3	86.2	60.3	**-**	101.4	92.7	83.8	**-**	99.7

¹time between first symptom presentation in primary care and diagnosis

²symptoms were categorized as alert or non-alert based on the NICE guidelines

### Excess mortality modelling

We found no evidence of an association between diagnostic interval and mortality for breast, colorectal and lung cancer (Table 6). There was some evidence that longer diagnostic intervals were associated with lower mortality amongst men with prostate cancer. There was also no evidence of an interaction between age and diagnostic interval on survival (p-values for interaction: breast = 0.35; colorectal = 0.31; lung = 0.13; prostate = 0.34).Associations between diagnostic interval and mortality for each cancer site varied in direction when stratified by classification of presenting symptom. We found no evidence of higher excess mortality with longer diagnostic intervals among women with breast cancer presenting with NICE-qualifying alert symptoms (p-values with diagnostic interval of <1 week as reference: 1–2 weeks = 0.850; 3–4 weeks = 0.055; >1 month = 0.346). There was some evidence that both shorter and longer diagnostic intervals were associated with decreased mortality for women who presented with non-alert symptoms, but multivariable analysis could not be done, as the models did not converge, due to the small number of deaths recorded in this group (n = 71). Among colorectal cancer patients presenting with NICE-qualifying alert symptoms, we observed higher mortality for both shorter and longer diagnostic intervals, but all results were imprecisely estimated (have wide confidence intervals; p-values with diagnostic interval of <1 month as reference: 1–2 months = 0.216; 3–6 months = 0.053; >6 months = 0.961). There was inconclusive evidence that a shorter diagnostic interval was associated with increased mortality among colorectal patients who presented with non-alert symptoms (p-values with diagnostic interval of <1 month as reference: 1–2 months = 0.493; 3–6 months = 0.349; >6 months = 0.049).There was little variation in the diagnostic interval—mortality associations among lung cancer patients who presented with NICE-qualifying symptoms (p-values with diagnostic interval of <1 month as reference: 1–2 months = 0.919; 3–6 months = 0.069; >6 months = 0.872). Among patients presenting with non-alert symptoms, there was some evidence that mortality was lower among patients who had diagnostic intervals of 3 months or more compared with those with shorter diagnostic intervals of less than 1 month (EHR 3–6 months vs <1 month: 0.81; 95% CI: 0.74–0.88; p-value = 0.001); EHR >6 months vs <1 month: 0.83; 95% CI: 0.76–0.90; p-value<0.001).Due to the high survival of patients with long diagnostic intervals among men with prostate cancer who presented with alert symptoms, excess hazards modelling could not be done (models did not converge). For men who presented with non-alert symptoms, there was evidence of decreasing mortality with longer diagnostic intervals (p-values with diagnostic interval of <1 month as reference: 1–2 months = 0.006; 3–6 months = 0.010; >6 months <0.001).Multivariate analysis for other variables is presented in S6 Table.

**Table 6:** Excess hazards models of cancer patients by site, diagnostic interval^1^ and symptom category^2^.

Cancer site / Diagnostic interval^1^	All Patients									Alert Symptoms									Non-alert Symptoms								
	Univariable				Multivariable					Univariable				Multivariable					Univariable				Multivariable				
	Excess Hazards Ratio	95% Confidence Interval			Excess Hazards Ratio	95% Confidence Interval			p-value	Excess Hazards Ratio	95% Confidence Interval			Excess Hazards Ratio	95% Confidence Interval			p-value	Excess Hazards Ratio	95% Confidence Interval			Excess Hazards Ratio	95% Confidence Interval			p-value
**Breast³**																											
* < 1 week*	1.00				1.00					1.00				1.00					1.00								
* 1–2 weeks*	1.14	0.93	**-**	1.39	1.04	0.86	**-**	1.25	0.674	1.12	0.91	**-**	1.38	1.02	0.84	**-**	1.23	0.850	3.01	0.36	**-**	25.54	Convergence not achieved; too few events				
* 3–4 weeks*	0.77	0.61	**-**	0.98	0.85	0.68	**-**	1.05	0.136	0.73	0.57	**-**	0.93	0.80	0.64	**-**	1.00	0.055	3.52	0.43	**-**	28.52					
* > 1 month*	0.73	0.59	**-**	0.92	0.88	0.71	**-**	1.09	0.234	0.77	0.61	**-**	0.96	0.90	0.73	**-**	1.12	0.346	1.21	0.15	**-**	9.55					
**Colorectal^4^**																											
* <1 month*	1.00				1.00					1.00				1.00					1.00				1.00				
* 1–2 months*	0.89	0.78	**-**	1.00	0.94	0.82	**-**	1.07	0.343	0.80	0.64	**-**	0.99	0.86	0.68	**-**	1.09	0.216	0.91	0.78	**-**	1.05	0.94	0.79	**-**	1.12	0.493
* 3–6 months*	0.83	0.74	**-**	0.93	0.92	0.82	**-**	1.04	0.174	0.71	0.57	**-**	0.87	0.80	0.64	**-**	1.00	0.053	0.79	0.69	**-**	0.90	0.93	0.80	**-**	1.08	0.349
* >6 months*	0.98	0.87	**-**	1.11	0.93	0.81	**-**	1.07	0.326	1.07	0.82	**-**	1.41	0.99	0.74	**-**	1.32	0.961	0.80	0.69	**-**	0.92	0.85	0.72	**-**	1.00	0.049
**Lung^5^**																											
* <1 month*	1.00				1.00					1.00				1.00					1.00				1.00				
* 1–2 months*	0.97	0.89	**-**	1.06	1.02	0.93	**-**	1.11	0.648	0.87	0.70	**-**	1.08	0.99	0.78	**-**	1.25	0.919	0.96	0.87	**-**	1.05	1.00	0.91	**-**	1.11	0.916
* 3–6 months*	0.84	0.78	**-**	0.91	0.90	0.84	**-**	0.98	0.014	0.71	0.56	**-**	0.90	0.79	0.62	**-**	1.02	0.069	0.81	0.74	**-**	0.88	0.87	0.80	**-**	0.95	0.001
* >6 months*	0.89	0.82	**-**	0.96	0.87	0.80	**-**	0.94	0.001	0.93	0.69	**-**	1.27	0.97	0.70	**-**	1.36	0.872	0.83	0.76	**-**	0.90	0.81	0.74	**-**	0.89	0.000
**Prostate^6^**																											
* <1 month*	1.00				1.00														1.00				1.00				
* 1–2 months*	0.49	0.33	**-**	0.73	0.64	0.42	**-**	0.98	0.042	0.52	0.17	**-**	1.63	Convergence not achieved; high survival in last group					0.45	0.30	**-**	0.69	0.51	0.32	**-**	0.82	0.006
* 3–6 months*	0.40	0.27	**-**	0.58	0.72	0.49	**-**	1.05	0.084	0.51	0.17	**-**	1.54						0.34	0.23	**-**	0.51	0.58	0.38	**-**	0.88	0.010
* >6 months*	0.16	0.07	**-**	0.38	0.45	0.27	**-**	0.75	0.002	0.38	0.07	**-**	2.03						0.12	0.05	**-**	0.33	0.37	0.21	**-**	0.64	0.000

¹time between first symptom presentation in primary care and diagnosis

²symptoms were categorized as alert or non-alert based on the NICE guidelines

³adjusted for age, sex, ethnicity, level of deprivation, morphology, tumour differentiation, number of consultations, treatment and comorbidity

^4^adjusted for age, sex, ethnicity, Duke stage, level of deprivation, morphology, tumour differentiation, tumour subsite, number of consultations, treatment and comorbidity

^5^adjusted for age, sex, ethnicity, tumour grade, smoking status, level of deprivation, morphology, number of consultations, treatment and comorbidity

^6^adjusted for age (15–54, 55–64, 64–74, 75+), ethnicity, tumour differentiation, level of deprivation, morphology, number of consultations, treatment and comorbidity

## Discussion

### Summary

Colorectal, lung and prostate cancer patients with NICE-qualifying alert symptoms had lower mortality compared to patients with non-alert symptoms, but findings were opposite for breast cancer. For colorectal and lung cancers, longer diagnostic intervals were associated with lower mortality for patients with non-alert symptoms. The risk of excess mortality in men with prostate cancer decreased with longer diagnostic intervals, regardless of symptom classification.

### Strengths and limitations

Our study is one of the few that have looked at the associations of diagnostic intervals with cancer survival, and even fewer studies have stratified by NICE-qualifying alert and non-alert symptom. We used secondary data from the CPRD, cancer registries, HES and ONS in England, which are databases known to be of high quality [25,50]. However, our study is not without limitations. We did not have pertinent information on all factors that could affect survival. Data on stage was only available for colorectal cancers and we had limited information on tumour differentiation for lung and prostate cancers. Nevertheless, we adjusted for treatment received (surgery, radiotherapy, chemotherapy and hormone therapy), which is a proxy indicator of disease severity, and we computed conditional relative survival estimates, to take into account factors influencing survival in the first year after diagnosis.Our study only included patients who consulted with a GP, representing those who are directly affected by the rapid diagnostic pathways specified in the cancer waiting time guidelines. The exclusion of patients diagnosed through emergency routes could have caused an underestimate of any observed association between short diagnostic intervals and increased mortality. Our findings would therefore only be strengthened by the inclusion of emergency presentations that bypassed the GP. Positive associations of diagnostic interval with mortality have been reported by other studies [15,17,19–22,51] and exclusion of emergency presentations alone was not sufficient to explain the results. The exclusion of patients diagnosed via screening could have resulted in an overestimate of the observed associations, since these patients tend to have short diagnostic intervals but better survival. However, their inclusion would have only affected breast and prostate cancer patients. It would also have resulted in a lead time bias that could have overestimated the excess hazards ratios, particularly for prostate cancer.Our estimates of diagnostic intervals were based on the date of the earliest recorded symptom within a period of one year prior to diagnosis. This symptom record could reflect the consultation when the GP thought the symptom might be related to cancer or the date when the patient was referred to secondary care for further investigation. There could be an underestimate of diagnostic interval, if the GP did not refer the patient on initial presentation or if they did not record the symptom until after making the decision to refer. The reported low proportion of GP consultation notes that were coded electronically [52] could have also influenced the measured diagnostic interval. Any underestimate would be more apparent for patients presenting with non-alert symptoms, which could be attributed to other diseases. Nevertheless, we believe this bias is non-differential with respect to survival and would underestimate observed associations.

### Comparison with previous studies

Our estimates of diagnostic intervals were shorter than previously reported using the CPRD [53]. The main difference lies in the classification of the symptoms as NICE-qualifying alert and non-alert. Some symptoms, classified as alert by Neal et al (diarrhoea for colorectal cancer; chest pain and cough for lung cancer), have been classified as non-alert in our study. We have also excluded some symptoms such as anaemia, anorexia, fatigue and weight loss for breast cancer from our list, as these were deemed too unspecific for the cancer site. We relied heavily on the referral guidelines and previous study definitions [31], and we felt that our classifications reflected the GP decision making process for referral.The findings that colorectal and lung cancer patients who presented with alert symptoms have better survival than those with non-alert symptoms were similar to a previous study using CPRD [16]. The disparities could be explained by the longer diagnostic interval for patients presenting with non-alert symptoms. Patients presenting with NICE-qualifying alert symptoms should have been referred to the rapid pathway, which would limit the time from referral to hospital appointment to two-weeks [7]. However, the same two-week pathway may increase the diagnostic interval for patients with non-alert symptoms [54], as indicated by the doubling of the diagnostic intervals for non-alert symptoms in our study.The associations between diagnostic interval and survival were masked when analyses combined all patients by cancer site, and did not stratify by symptom. In recent UK studies, no associations between longer diagnostic interval and higher mortality were found for lung and colorectal cancers, where all patients were combined in the analysis per cancer site [16,55]. For colorectal cancer, one study showed high mortality for patients with short and long diagnostic intervals in the unadjusted regression analysis but the association was attenuated once tumour biology was taken into account [55]. We, however, found that the associations between diagnostic interval and survival differ by site and symptom classification, even after tumour biology was taken into account.In a previous study in Denmark, the highest mortality rates were observed for those with both the shortest and longest diagnostic intervals among patients who presented with symptoms suggestive of cancer [21], conversely, for patients presenting with vague symptoms, the lowest mortality rates were seen among those with the shortest and longest diagnostic intervals [21]. While our study showed similar associations for colorectal and lung cancer patients with alert symptoms, our results only show lower mortality with longer diagnostic intervals for non-alert symptoms [21]. Our dataset enabled us to adjust for confounding variables such as tumour differentiation, and to some extent, stage, which were not taken into account in the previous study.Some of our findings appear to run counter to expected associations: high mortality among patients with short diagnostic intervals and low mortality for patients with long diagnostic intervals. These associations remained even after adjustments for tumour biology, and could be attributed to confounding by indication. Confounding by indication is an extraneous determinant of the outcome parameter that is present if a perceived high risk or poor prognosis becomes an indication for intervention [56]. In our study, the type of presenting symptoms (alert or non-alert) could have triggered differences in care. Patients presenting with severe manifestations of cancer could be expedited through the system [57], with the result that these patients have shorter diagnostic interval yet show higher mortality [16,19–22,51]. More research is needed to elucidate how the role of health care affects diagnostic intervals and survival.The higher mortality observed for colorectal cancer patients with shorter and longer diagnostic intervals is in agreement with existing literature [12,21,22] and reinforces the rationale for rapid diagnostic pathways. Patients with poorer prognosis could have shorter diagnostic intervals because they were expedited through the pathway. Patients with longer diagnostic intervals could have suffered from delays that might have resulted in disease progression [11] which could have led to an adverse effect on survival [11,21]. Longer diagnostic intervals could have been a result of patient delays, increased burden to secondary care, or longer diagnostic work up [13,58,59].There is a possibility that time from diagnosis to treatment might have contributed to excess mortality, with longer waiting times for treatment worsening disease prognosis. Current evidence regarding this is inconclusive [23,60], with some studies on breast, lung and colorectal cancers showing no association or some evidence of the waiting time paradox [23,60]. Based on the literature we believe that any residual confounding would have been minimal and will not alter the results of our study.

### Implications for practice

Despite our finding, clinicians should be mindful that whatever the association between presenting symptoms and mortality, perceived delayed diagnoses can have negative effects for both the psychological health of the patient and on the patient-doctor relationship. GPs should continue to refer patients with alert symptoms via the cancer pathways, and at least actively follow-up patients with non-alert symptoms in the community. Nevertheless, our study provides some reassurance for patients and clinicians alike that a reasonable diagnostic interval should not worsen prognosis and decrease survival.

### Conclusions

The disparate effects of diagnostic intervals on the excess mortality of patients with alert and non-alert symptoms highlight the importance of the nature of the symptoms and type of cancer. The findings in this and other studies suggest that alert symptoms may prompt earlier diagnosis, not only by drawing attention to the underlying cancer, but also by prompting immediate action from clinicians. However, the UK’s two-week pathway may increase the diagnostic interval for patients with non-alert symptoms.

## Supporting Information

S1 Table Distribution of cancer patients by site specific characteristics (tumour morphology, subsite, staging and smoking status). (DOCX)

S2 Table Presenting symptoms of breast cancer patients and diagnostic interval. (DOCX)

S3 Table Presenting symptoms of colorectal cancer patients and diagnostic interval. (DOCX)

S4 Table Presenting symptoms of lung cancer patients and diagnostic interval. (DOCX)

S5 Table Presenting symptoms of prostate cancer patients and diagnostic interval. (DOCX)

S6 Table Multivariable analysis for covariables. (DOCX)

## References

[1] Coleman MP, Forman D, Bryant H, Butler J, Rachet B, Maringe C, et al Cancer survival in Australia, Canada, Denmark, Norway, Sweden, and the UK, 1995–2007 (the International Cancer Benchmarking Partnership): an analysis of population-based cancer registry data. Lancet. 2011 377: 127–138. 10.1016/S0140-6736(10)62231-3 21183212 PMC3018568

[2] Holmberg L, Robinson D, Sandin F, Bray F, Linklater KM, Klint A, et al A comparison of prostate cancer survival in England, Norway and Sweden: a population-based study. Cancer Epidemiol. 2012 36: e7–12. 10.1016/j.canep.2011.08.001 21907655

[3] De Angelis R, Sant M, Coleman MP, Francisci S, Baili P, Pierannunzio D, et al Cancer survival in Europe 1999–2007 by country and age: results of EUROCARE-5-a population-based study. Lancet Oncol. 2014 15: 23–34. 10.1016/S1470-2045(13)70546-1 24314615

[4] Richards MA. The size of the prize for earlier diagnosis of cancer in England. Br J Cancer. 2009 101 Suppl 2: S125–129. 10.1038/sj.bjc.6605402 19956156 PMC2790715

[5] Department of Health. The NHS Cancer Plan. London: Department of Health 2000.

[6] Department of Health. Cancer Reform Strategy. London: Department of Health 2007.

[7] National Institute for Health and Clinical Excellence. Referral guidelines for suspected cancer London: National Institute for Health and Clinical Excellence 2005.

[8] National Institute for Health and Clinical Excellence. Referral guidelines for suspected cancer. 2014. [cited March 13, 2015]. Available: https://www.nice.org.uk/guidance/cg27.

[9] Neal RD, Allgar VL. Sociodemographic factors and delays in the diagnosis of six cancers: analysis of data from the "National Survey of NHS Patients: Cancer". Br J Cancer. 2005 92: 1971–1975. 15900296 10.1038/sj.bjc.6602623 PMC2361785

[10] Department of Health, Public Health England, NHS England. Improving outcomes: a strategy for cancer- third annual report. London: Department of Health.2013; pp.

[11] Richards MA, Smith P, Ramirez AJ, Fentiman IS, Rubens RD. The influence on survival of delay in the presentation and treatment of symptomatic breast cancer. Br J Cancer. 1999 79: 858–864. 10070881 10.1038/sj.bjc.6690137 PMC2362673

[12] Torring ML, Frydenberg M, Hamilton W, Hansen RP, Lautrup MD, Vedsted P. Diagnostic interval and mortality in colorectal cancer: U-shaped association demonstrated for three different datasets. J Clin Epidemiol. 2012 65: 669–678. 10.1016/j.jclinepi.2011.12.006 22459430

[13] Porta M, Gallen M, Malats N, Planas J. Influence of "diagnostic delay" upon cancer survival: an analysis of five tumour sites. J Epidemiol Community Health. 1991 45: 225–230. 1757766 10.1136/jech.45.3.225 PMC1060763

[14] Richards MA, Westcombe AM, Love SB, Littlejohns P, Ramirez AJ. Influence of delay on survival in patients with breast cancer: a systematic review. Lancet. 1999 353: 1119–1126. 10209974 10.1016/s0140-6736(99)02143-1

[15] Ramos M, Esteva M, Cabeza E, Campillo C, Llobera J, Aguilo A. Relationship of diagnostic and therapeutic delay with survival in colorectal cancer: a review. Eur J Cancer. 2007 43: 2467–2478. 17931854 10.1016/j.ejca.2007.08.023

[16] Dregan A, Møller H, Charlton J, Gulliford M. Are alarm symptoms predictive of cancer survival? Br J Gen Pract.2013; 63: e807–812. 10.3399/bjgp13X675197 24351496 PMC3839389

[17] Rupassara KS, Ponnusamy S, Withanage N, Milewski PJ. A paradox explained? Patients with delayed diagnosis of symptomatic colorectal cancer have good prognosis. Colorectal Dis. 2006 8: 423–429. 16684087 10.1111/j.1463-1318.2006.00958.x

[18] Terhaar sive Droste JS, Oort FA, van der Hulst RW, Coupe VM, Craanen ME, Meijer GA, et al Does delay in diagnosing colorectal cancer in symptomatic patients affect tumor stage and survival? A population-based observational study. BMC Cancer. 2010 10: 332 10.1186/1471-2407-10-332 20584274 PMC2907342

[19] Gonzalez-Barcala FJ, Garcia-Prim JM, Alvarez-Dobano JM, Moldes-Rodriguez M, Garcia-Sanz MT, Pose-Reino A, et al Effect of delays on survival in patients with lung cancer. Clin Transl Oncol. 2010 12: 836–842. 10.1007/s12094-010-0606-5 21156415

[20] Radzikowska E, Roszkowski-Sliz K, Chabowski M, Glaz P. Influence of delays in diagnosis and treatment on survival in small cell lung cancer patients. Adv Exp Med Biol. 2013 788: 355–362. 10.1007/978-94-007-6627-3_48 23835998

[21] Torring ML, Frydenberg M, Hansen RP, Olesen F, Vedsted P. Evidence of increasing mortality with longer diagnostic intervals for five common cancers: A cohort study in primary care. Eur J Cancer. 2013. 10.1016/j.ejca.2013.01.025 23453935

[22] Pruitt SL, Harzke AJ, Davidson NO, Schootman M. Do diagnostic and treatment delays for colorectal cancer increase risk of death? Cancer Causes Control. 2013 24: 961–977. 10.1007/s10552-013-0172-6 23446843 PMC3708300

[23] Neal RD, Tharmanathan P, France B, Din NU, Cotton S, Fallon-Ferguson J, et al. Is increased time to diagnosis and treatment in symptomatic cancer associated with poorer outcomes? Systematic review. Br J Cancer. 2015. 10.1038/bjc.2015.48 PMC4385982 25734382

[24] Neal RD. Do diagnostic delays in cancer matter? Br J Cancer. 2009 101 Suppl 2: S9–S12. 10.1038/sj.bjc.6605384 19956171 PMC2790696

[25] Dregan A, Moller H, Murray-Thomas T, Gulliford MC. Validity of cancer diagnosis in a primary care database compared with linked cancer registrations in England. Population-based cohort study. Cancer Epidemiol. 2012. 10.1016/j.canep.2012.05.013 22727737

[26] Herrett E, Thomas SL, Schoonen WM, Smeeth L, Hall AJ. Validation and validity of diagnoses in the General Practice Research Database: a systematic review. Br J Clin Pharmacol. 2010 69: 4–14. 10.1111/j.1365-2125.2009.03537.x 20078607 PMC2805870

[27] Khan NF, Harrison SE, Rose PW. Validity of diagnostic coding within the General Practice Research Database: a systematic review. Br J Gen Pract. 2010 60: e128–136. 10.3399/bjgp10X483562 20202356 PMC2828861

[28] Kaye JA, Derby LE, del Mar Melero-Montes M, Quinn M, Jick H. The incidence of breast cancer in the General Practice Research Database compared with national cancer registration data. Br J Cancer. 2000 83: 1556–1558. 11076668 10.1054/bjoc.2000.1493 PMC2363417

[29] CPRD. Cancer Registry data and CPRD London: Clinical Practice Research Datalink 2012.

[30] Noble M, mcLennan D, Wilkinson K, Whitworth A, Barnes H, Dibben C. The English Indices of Deprivation 2007. London: Communities and Local Government 2008.

[31] Hamilton W. The CAPER studies: five case-control studies aimed at identifying and quantifying the risk of cancer in symptomatic primary care patients. Br J Cancer. 2009 101 Suppl 2: S80–86. 10.1038/sj.bjc.6605396 19956169 PMC2790706

[32] European Network of Cancer Registries. ENCR Definitions for the coding of basis of diagnosis. Lyon, France: ENCR. 1997.

[33] Weller D, Vedsted P, Rubin G, Walter FM, Emery J, Scott S, et al The Aarhus statement: improving design and reporting of studies on early cancer diagnosis. Br J Cancer. 2012 106: 1262–1267. 10.1038/bjc.2012.68 22415239 PMC3314787

[34] Department of Health. A Practical Guide to Ethnic Monitoring in the NHS and Social Care. London: Department of Health 2005.

[35] Hospital Episode Statistics. How good is HES ethnic coding and where do the problems lie? London: The Health and Social Care Information Centre 2009.

[36] Office of the Deputy Prime Minister. The English Indices of Deprivation 2004: Summary. London: Office of the Deputy Prime Minister 2004.

[37] Tavassoli F, Devilee P, editors. Pathology and Genetics of Tumours of the Breast and Female Genital Organs. Lyon: IARC; 2003.

[38] Bosman F, Carneiro F, Hruban R, Theise N, editors. Pathology and Genetics Tumours of the Digestive System: IARC; 2010.

[39] Travis WD, Brambilla E, Müller-Hermelink HK, Harris CC, editors. Pathology and Genetics of Tumours of the Lung, Pleura, Thymus and Heart. Lyon: IARC; 2004.

[40] Eble J, Sauter G, Epstein J, Sesterhenn I, editors. Pathology and Genetics of Tumours of the Urinary System and Male Genital Organs. Lyon: IARC; 2004.

[41] Charlson ME, Pompei P, Ales KL, MacKenzie CR. A new method of classifying prognostic comorbidity in longitudinal studies: development and validation. J Chronic Dis. 1987 40: 373–383. 3558716 10.1016/0021-9681(87)90171-8

[42] Ederer F, Axtell LM, Cutler SJ. The relative survival rate: a statistical methodology. Natl Cancer Inst Monogr. 1961 6: 101–121. 13889176

[43] Brenner H, Gefeller O, Hakulinen T. Period analysis for 'up-to-date' cancer survival data: theory, empirical evaluation, computational realisation and applications. Eur J Cancer. 2004 40: 326–335. 14746849 10.1016/j.ejca.2003.10.013

[44] Cancer Research UK Cancer Survival Group. Life tables for England by sex, calendar period, region and deprivation London School of Hygiene & Tropical Medicine 2009; pp.

[45] Dickman PW, Sloggett A, Hills M, Hakulinen T. Regression models for relative survival. Stat Med. 2004 23: 51–64. 14695639 10.1002/sim.1597

[46] Nur U, Shack LG, Rachet B, Carpenter JR, Coleman MP. Modelling relative survival in the presence of incomplete data: a tutorial. Int J Epidemiol. 2010 39: 118–128. 10.1093/ije/dyp309 19858106

[47] Royston P. Multiple imputation of missing values: Update of ice The Stata Journal 2005 5: 527–536.

[48] Sterne JA, White IR, Carlin JB, Spratt M, Royston P, Kenward MG, et al Multiple imputation for missing data in epidemiological and clinical research: potential and pitfalls. BMJ. 2009 338: b2393 10.1136/bmj.b2393 19564179 PMC2714692

[49] StataCorp. Stata Statistical Software: Release 12. College Station, TX: StataCorp LP 2011; pp.

[50] Office of National Statistics. Cancer Registrations Statistics, England, 2011. 2013. [cited 19 September, 2014]. Available: http://www.ons.gov.uk/ons/rel/vsob1/cancer-statistics-registrations—england—series-mb1-/no—42—2011/stb-cancer-statistics-registrations-2011.html#tab-Cancer-registrations—interpretation-and-data-quality.

[51] Myrdal G, Lambe M, Hillerdal G, Lamberg K, Agustsson T, Stahle E. Effect of delays on prognosis in patients with non-small cell lung cancer. Thorax. 2004 59: 45–49. 14694247 PMC1758865

[52] Salisbury C, Procter S, Stewart K, Bowen L, Purdy S, Ridd M, et al The content of general practice consultations: cross-sectional study based on video recordings. Br J Gen Pract. 2013 63: e751–759. 10.3399/bjgp13X674431 24267858 PMC3809428

[53] Neal RD, Din NU, Hamilton W, Ukoumunne OC, Carter B, Stapley S, et al Comparison of cancer diagnostic intervals before and after implementation of NICE guidelines: analysis of data from the UK General Practice Research Database. Br J Cancer. 2014 110: 584–592. 10.1038/bjc.2013.791 24366304 PMC3915139

[54] Rai S, Kelly MJ. Prioritization of colorectal referrals: a review of the 2-week wait referral system. Colorectal Dis. 2007 9: 195–202. 17298615 10.1111/j.1463-1318.2006.01107.x

[55] Murchie P, Raja EA, Brewster DH, Campbell NC, Ritchie LD, Robertson R, et al Time from first presentation in primary care to treatment of symptomatic colorectal cancer: effect on disease stage and survival. Br J Cancer. 2014 111: 461–469. 10.1038/bjc.2014.352 24992583 PMC4119995

[56] OS M. Theoretical epidemiology: principles of occurence research in medicine Ney York: John Wiley & Sons; 1985.

[57] Hamilton W. Cancer diagnosis in primary care. Br J Gen Pract. 2010 60: 121–128. 10.3399/bjgp10X483175 20132704 PMC2814263

[58] Korsgaard M, Pedersen L, Sorensen HT, Laurberg S. Reported symptoms, diagnostic delay and stage of colorectal cancer: a population-based study in Denmark. Colorectal Dis. 2006 8: 688–695. 16970580 10.1111/j.1463-1318.2006.01014.x

[59] Williams JG, Roberts SE, Ali MF, Cheung WY, Cohen DR, Demery G, et al Gastroenterology services in the UK. The burden of disease, and the organisation and delivery of services for gastrointestinal and liver disorders: a review of the evidence. Gut. 2007 56 Suppl 1: 1–113. 17303614 10.1136/gut.2006.117598 PMC1860005

[60] Redaniel MT, Martin RM, Blazeby JM, Wade J, Jeffreys M. The association of time between diagnosis and major resection with poorer colorectal cancer survival: a retrospective cohort study. BMC Cancer. 2014 14: 642 10.1186/1471-2407-14-642 25175937 PMC4159515

