# Supplementary material for: Diagnostic Intervals and Its Association with Breast, Prostate, Lung and Colorectal Cancer Survival in England: Historical Cohort Study Using the Clinical Practice Research Datalink
Source: PLoS One. 2015 May 1;10(5):e0126608. doi: 10.1371/journal.pone.0126608 (PMC4416709; doi:10.1371/journal.pone.0126608)
Supplement: S1 Table — (DOCX) [file pone.0126608.s001.docx]

| **S1 Table. Distribution of cancer patients by site specific characteristics**  **(tumour morphology, subsite, staging and smoking status)** | | | |
| --- | --- | --- | --- |
|  |  |  | |
| **Site** | **Freq.** | | **%** |
| **Breast** |  | |  |
| **Tumour morphology** |  | |  |
| *Invasive ductal carcinoma* | 6,453 | | 74.7 |
| *Invasive lobular carcinoma* | 924 | | 10.7 |
| *other* | 1,202 | | 13.9 |
| *unknown* | 60 | | 0.7 |
| **Colorectal** |  | |  |
| **Tumour morphology** |  | |  |
| *Adenocarcinoma* | 4,782 | | 80.9 |
| *Mucinous adenocarcinoma* | 273 | | 4.6 |
| *other* | 760 | | 12.9 |
| *unknown* | 97 | | 1.6 |
| **Subsite** |  | |  |
| *Colon* | 3,406 | | 57.6 |
| *Rectosigmoid* | 538 | | 9.1 |
| *Rectum* | 1,968 | | 33.3 |
| **Dukes’ stage** |  | |  |
| *A* | 490 | | 8.3 |
| *B* | 1,442 | | 24.4 |
| *C* | 1,686 | | 28.5 |
| *D* | 549 | | 9.3 |
| *unknown* | 1,745 | | 29.5 |
| **Lung** |  | |  |
| **Tumour morphology** |  | |  |
| *Squamous cell carcinoma* | 1,362 | | 23.7 |
| *Small cell carcinoma* | 762 | | 13.3 |
| *Adenocarcinoma* | 995 | | 17.3 |
| *Large cell carcinoma* | 106 | | 1.9 |
| *Other* | 2,234 | | 38.9 |
| *unknown* | 278 | | 4.9 |
| **Smoking status** |  | |  |
| *Non-smoker* | 615 | | 10.7 |
| *Current smoker* | 2,178 | | 38.0 |
| *Ex-smoker* | 2,320 | | 40.4 |
| *Unknown* | 624 | | 10.9 |
| **Prostate** |  | |  |
| **Tumour morphology** |  | |  |
| *Adenocarcinoma* | 1,606 | | 91.1 |
| *other* | 124 | | 7.0 |
| *unknown* | 33 | | 1.9 |
