# Supplementary material for: Diagnostic Intervals and Its Association with Breast, Prostate, Lung and Colorectal Cancer Survival in England: Historical Cohort Study Using the Clinical Practice Research Datalink
Source: PLoS One. 2015 May 1;10(5):e0126608. doi: 10.1371/journal.pone.0126608 (PMC4416709; doi:10.1371/journal.pone.0126608)
Supplement: S2 Table — (DOCX) [file pone.0126608.s002.docx]

| **S2 Table. Presenting symptoms of breast cancer patients and diagnostic interval** | | | | | | | | |
| --- | --- | --- | --- | --- | --- | --- | --- | --- |
| **Category/Symptom** | **N** | **Median** | **IQR** | | | **Range** | | |
| **Overall** | **8,639** | **14** | **9** | **-** | **31** | **1** | **-** | **365** |
| **Alert** | **8,150** | **14** | **9** | **-** | **29** | **1** | **-** | **365** |
| *Bloody nipple* | 1 | 20 | 20 | **-** | 20 | 20 | **-** | 20 |
| *Breast lump* | 7,988 | 14 | 9 | **-** | 28 | 1 | **-** | 365 |
| *Breast skin changes* | 14 | 10 | 8 | **-** | 42 | 6 | **-** | 224 |
| *Nipple distortion* | 127 | 21 | 12 | **-** | 42 | 2 | **-** | 287 |
| *Nipple eczema* | 20 | 26 | 11 | **-** | 63.5 | 1 | **-** | 341 |
| **Non-alert** | **489** | **55** | **21** | **-** | **175** | **1** | **-** | **365** |
| *Breast pain* | 394 | 56 | 22 | **-** | 166.0 | 1 | **-** | 365 |
| *Family history* | 95 | 42 | 12 | **-** | 216 | 1 | **-** | 361 |
