# Supplementary material for: Diagnostic Intervals and Its Association with Breast, Prostate, Lung and Colorectal Cancer Survival in England: Historical Cohort Study Using the Clinical Practice Research Datalink
Source: PLoS One. 2015 May 1;10(5):e0126608. doi: 10.1371/journal.pone.0126608 (PMC4416709; doi:10.1371/journal.pone.0126608)
Supplement: S3 Table — (DOCX) [file pone.0126608.s003.docx]

| **S3 Table. Presenting symptoms of colorectal cancer patients and diagnostic interval** | | | | | | | | |
| --- | --- | --- | --- | --- | --- | --- | --- | --- |
| **Category/Symptom** | **N** | **Median** | **IQR** | | | **Range** | | |
| **Overall** | **5,912** | **67** | **27** | **-** | **147** | **1** | **-** | **365** |
| **Alert** | **2,178** | **45** | **20** | **-** | **95** | **1** | **-** | **365** |
| *Change in bowel habit* | 809 | 45 | 21 | **-** | 91 | 2 | **-** | 352 |
| *Rectal bleeding* | 1,224 | 47.5 | 21 | **-** | 104 | 1 | **-** | 365 |
| *Palpable mass* | 145 | 30 | 14 | **-** | 69 | 1 | **-** | 346 |
| **Non-alert** | **3,734** | **84** | **34** | **-** | **186** | **1** | **-** | **365** |
| *Abdominal pain* | 1,655 | 84 | 33 | **-** | 175 | 1 | **-** | 365 |
| *Anorexia* | 45 | 87 | 37 | **-** | 240 | 8 | **-** | 347 |
| *Constipation* | 679 | 92 | 30 | **-** | 209 | 1 | **-** | 364 |
| *Diarrhoea* | 879 | 76 | 34 | **-** | 170 | 1 | **-** | 364 |
| *Fatigue* | 271 | 121 | 49 | **-** | 239 | 2 | **-** | 365 |
| *Weight loss* | 205 | 76 | 33 | **-** | 168 | 3 | **-** | 359 |
