# Supplementary material for: Diagnostic Intervals and Its Association with Breast, Prostate, Lung and Colorectal Cancer Survival in England: Historical Cohort Study Using the Clinical Practice Research Datalink
Source: PLoS One. 2015 May 1;10(5):e0126608. doi: 10.1371/journal.pone.0126608 (PMC4416709; doi:10.1371/journal.pone.0126608)
Supplement: S4 Table — (DOCX) [file pone.0126608.s004.docx]

| **S4 Table. Presenting symptoms of lung cancer patients and diagnostic interval** | | | | | | | | |
| --- | --- | --- | --- | --- | --- | --- | --- | --- |
| **Category/Symptom** | **N** | **Median** | **IQR** | | | **Range** | | |
| **Overall** | **5,737** | **88** | **34** | **-** | **210** | **1** | **-** | **365** |
| **Alert** | **533** | **35** | **17** | **-** | **78** | **1** | **-** | **362** |
| *Haemoptysis* | 506 | 35 | 17 | **-** | 78 | 1 | **-** | 362 |
| *SVC obstruction* | 24 | 30.5 | 12 | **-** | 142 | 3 | **-** | 355 |
| *Stridor* | 3 | 65 | 8 | **-** | 311 | 8 | **-** | 311 |
| **Non-alert** | **5,204** | **99** | **38** | **-** | **222** | **1** | **-** | **365** |
| *Anorexia* | 95 | 40 | 23 | **-** | 120 | 2 | **-** | 359 |
| *Cervical lymphadenopathy* | 31 | 32 | 13 | **-** | 55 | 1 | **-** | 351 |
| *Chest signs* | 158 | 97 | 35 | **-** | 218 | 3 | **-** | 360 |
| *Chest/rib pain* | 857 | 92 | 37 | **-** | 196 | 1 | **-** | 365 |
| *Cough* | 2,346 | 114 | 44 | **-** | 241 | 1 | **-** | 365 |
| *Dyspnoea* | 1,061 | 71 | 26 | **-** | 196 | 1 | **-** | 365 |
| *Fatigue* | 234 | 119 | 51 | **-** | 231 | 8 | **-** | 365 |
| *Finger clubbing* | 28 | 58.5 | 35.5 | **-** | 113 | 8 | **-** | 306 |
| *Hoarseness* | 110 | 49 | 27 | **-** | 129 | 1 | **-** | 356 |
| *Shoulder pain* | 284 | 146.5 | 66.5 | **-** | 260 | 6 | **-** | 365 |
