# Supplementary material for: Diagnostic Intervals and Its Association with Breast, Prostate, Lung and Colorectal Cancer Survival in England: Historical Cohort Study Using the Clinical Practice Research Datalink
Source: PLoS One. 2015 May 1;10(5):e0126608. doi: 10.1371/journal.pone.0126608 (PMC4416709; doi:10.1371/journal.pone.0126608)
Supplement: S5 Table — (DOCX) [file pone.0126608.s005.docx]

| **S5 Table. Presenting symptoms of prostate cancer patients and diagnostic interval** | | | | | | | | |
| --- | --- | --- | --- | --- | --- | --- | --- | --- |
| **Category/Symptom** | **N** | **Median** | **IQR** | | | **Range** | | |
| **Overall** | **1,763** | **71** | **35** | **-** | **145** | **1** | **-** | **365** |
| **Alert** | **357** | **48** | **24** | **-** | **111** | **1** | **-** | **364** |
| *Enlarged prostate* | 357 | 48 | 24 | - | 111 | 1 | - | 364 |
| **Non-alert** | **1,406** | **76** | **37** | **-** | **151** | **1** | **-** | **365** |
| *Haematuria* | 593 | 82 | 38 | - | 163 | 1 | - | 364 |
| *Hesitancy* | 84 | 81.5 | 47.5 | - | 128 | 10 | - | 361 |
| *Nocturia* | 656 | 71.5 | 36 | - | 144 | 1 | - | 365 |
| *Poor stream* | 37 | 81 | 45 | - | 162 | 12 | - | 357 |
| *Terminal dribbling* | 36 | 69 | 35 | - | 160.5 | 5 | - | 359 |
