# Supplementary material for: Diagnostic Intervals and Its Association with Breast, Prostate, Lung and Colorectal Cancer Survival in England: Historical Cohort Study Using the Clinical Practice Research Datalink
Source: PLoS One. 2015 May 1;10(5):e0126608. doi: 10.1371/journal.pone.0126608 (PMC4416709; doi:10.1371/journal.pone.0126608)
Supplement: S6 Table — (DOCX) [file pone.0126608.s006.docx]

| **S6 Table. Multivariable analysis for covariables** | | | | | | | | | | | | | | | |  |  |
| --- | --- | --- | --- | --- | --- | --- | --- | --- | --- | --- | --- | --- | --- | --- | --- | --- | --- |
|  |  |  |  |  |  |  |  |  |  | |  |  |  |  |  |  |  |
| **Variable** | **Breast** | | | | **Colorectal** | | | | | **Lung** | | | | **Prostate** | | | |
|  | **Excess Hazards Ratio** | **95% Confidence Interval** | | | **Excess Hazards Ratio** | **95% Confidence Interval** | | | | **Excess Hazards Ratio** | **95% Confidence Interval** | | | **Excess Hazards Ratio** | **95% Confidence Interval** | | |
| **Age group (at diagnosis)** | | | | | | | | | | | | | | | | | |
| *15-44* | 1.00 |  |  |  | 1.00 |  |  |  | | 1.00 |  |  |  |  |  |  |  |
| *45-54* | 1.10 | 0.89 | - | 1.35 | 1.01 | 0.74 | - | 1.38 | | 0.80 | 0.62 | - | 1.03 | 1.00^1^ |  |  |  |
| *55-64* | 1.38 | 1.12 | - | 1.72 | 1.01 | 0.76 | - | 1.35 | | 0.86 | 0.67 | - | 1.09 | 1.19 | 0.50 | - | 2.82 |
| *65-74* | 1.67 | 1.31 | - | 2.12 | 1.08 | 0.82 | - | 1.43 | | 0.94 | 0.74 | - | 1.20 | 1.22 | 0.53 | - | 2.84 |
| *75 and above* | 2.73 | 2.12 | - | 3.53 | 1.28 | 0.97 | - | 1.70 | | 1.06 | 0.83 | - | 1.34 | 1.67 | 0.71 | - | 3.91 |
| **Sex** |  |  |  |  |  |  |  |  | |  |  |  |  |  |  |  |  |
| *Male* |  |  |  |  | 1.00 |  |  |  | | 1.00 |  |  |  |  |  |  |  |
| *Female* |  |  |  |  | 1.00 | 0.91 | - | 1.09 | | 0.94 | 0.89 | - | 1.00 |  |  |  |  |
| **Ethnicity** |  |  |  |  |  |  |  |  | |  |  |  |  |  |  |  |  |
| *White* | 1.00 |  |  |  | 1.00 |  |  |  | | 1.00 |  |  |  | 1.00 |  |  |  |
| *Black* | 1.44 | 0.85 | - | 2.45 | 1.15 | 0.64 | - | 2.06 | | 1.19 | 0.65 | - | 2.20 | 0.95 | 0.23 | - | 3.89 |
| *Asian* | 1.16 | 0.58 | - | 2.33 | 0.84 | 0.43 | - | 1.62 | | 0.83 | 0.49 | - | 1.43 | 0.64 | 0.04 | - | 11.42 |
| *Mixed^2^* | 0.26 | 0.02 | - | 3.75 | 0.27 | 0.03 | - | 2.40 | |  |  |  |  |  |  |  |  |
| *Other* | 1.13 | 0.62 | - | 2.03 | 1.06 | 0.53 | - | 2.12 | | 1.26 | 0.89 | - | 1.81 | 1.74 | 0.43 | - | 7.11 |
| *Unknown* | 1.07 | 0.90 | - | 1.26 | 1.12 | 1.00 | - | 1.26 | | 0.92 | 0.86 | - | 0.99 | 0.84 | 0.57 | - | 1.23 |
| **Region** |  |  |  |  |  |  |  |  | |  |  |  |  |  |  |  |  |
| *London* | 1.00 |  |  |  | 1.00 |  |  |  | | 1.00 |  |  |  | 1.00 |  |  |  |
| *North East* | 1.53 | 0.94 | - | 2.49 | 0.58 | 0.41 | - | 0.83 | | 0.80 | 0.66 | - | 0.96 | 1.70 | 0.51 | - | 5.69 |
| *North West* | 1.03 | 0.79 | - | 1.36 | 0.69 | 0.57 | - | 0.83 | | 0.81 | 0.72 | - | 0.92 | 1.79 | 0.89 | - | 3.59 |
| *Yorkshire and the Humber* | 1.26 | 0.89 | - | 1.77 | 0.68 | 0.53 | - | 0.89 | | 0.91 | 0.78 | - | 1.06 | 1.61 | 0.74 | - | 3.51 |
| *East Midlands* | 0.79 | 0.51 | - | 1.23 | 0.62 | 0.47 | - | 0.82 | | 0.77 | 0.65 | - | 0.92 | 0.50 | 0.11 | - | 2.24 |
| *West Midlands* | 1.14 | 0.85 | - | 1.55 | 0.86 | 0.69 | - | 1.07 | | 0.97 | 0.84 | - | 1.11 | 0.75 | 0.34 | - | 1.65 |
| *East of England* | 0.93 | 0.70 | - | 1.24 | 0.58 | 0.46 | - | 0.72 | | 0.83 | 0.73 | - | 0.94 | 0.83 | 0.38 | - | 1.82 |
| *South East* | 0.95 | 0.73 | - | 1.24 | 0.80 | 0.66 | - | 0.97 | | 0.93 | 0.83 | - | 1.05 | 1.08 | 0.55 | - | 2.10 |
| *South West* | 0.88 | 0.65 | - | 1.18 | 0.63 | 0.51 | - | 0.78 | | 0.84 | 0.73 | - | 0.96 | 1.13 | 0.53 | - | 2.40 |
| **Level of Deprivation** |  |  |  |  |  |  |  |  | |  |  |  |  |  |  |  |  |
| *1 - least derpived* | 1.00 |  |  |  | 1.00 |  |  |  | | 1.00 |  |  |  | 1.00 |  |  |  |
| *2* | 1.01 | 0.84 | - | 1.22 | 1.08 | 0.95 | - | 1.24 | | 1.08 | 0.98 | - | 1.19 | 0.86 | 0.57 | - | 1.29 |
| *3* | 0.91 | 0.74 | - | 1.13 | 1.14 | 0.99 | - | 1.32 | | 1.02 | 0.92 | - | 1.12 | 1.06 | 0.68 | - | 1.65 |
| *4* | 1.13 | 0.92 | - | 1.39 | 1.17 | 1.01 | - | 1.36 | | 1.04 | 0.95 | - | 1.15 | 1.09 | 0.68 | - | 1.75 |
| *5 - most deprived* | 1.20 | 0.95 | - | 1.52 | 1.42 | 1.20 | - | 1.67 | | 1.07 | 0.97 | - | 1.19 | 1.54 | 0.94 | - | 2.54 |
| **Period of Cancer Plan Implementation** | | | | | | | | | | | | | | | | | |
| *Prior to implementation* | 1.00 |  |  |  | 1.00 |  |  |  | | 1.00 |  |  |  | 1.00 |  |  |  |
| *Initialization* | 0.65 | 0.51 | - | 0.82 | 0.98 | 0.85 | - | 1.14 | | 1.22 | 1.12 | - | 1.33 | 0.51 | 0.34 | - | 0.78 |
| *Implementation* | 1.02 | 0.86 | - | 1.21 | 1.14 | 0.99 | - | 1.32 | | 1.32 | 1.20 | - | 1.45 | 0.89 | 0.56 | - | 1.41 |

^1^because of small numbers, the reference category was 15-54 age group

^2^too few number of cases to include in the model
